# Preoperative Sarcopenia Severity and Clinical Outcomes after Total Hip Arthroplasty

**DOI:** 10.3390/nu16132085

**Published:** 2024-06-29

**Authors:** Shinya Tanaka, Azusa Kayamoto, Chiaki Terai, Shusuke Nojiri, Yuki Fugane, Tomohiro Mori, Motoki Nagaya, Masato Kako, Hiroki Iida, Yusuke Osawa, Yasuhiko Takegami, Yoshihiro Nishida

**Affiliations:** 1 Department of Rehabilitation, Nagoya University Hospital, Nagoya 466-8560, Japan; s-tanaka@med.nagoya-u.ac.jp (S.T.); kayamoto@med.nagoya-u.ac.jp (A.K.); cterai@med.nagoya-u.ac.jp (C.T.); nojiris@med.nagoya-u.ac.jp (S.N.); fugane@med.nagoya-u.ac.jp (Y.F.); mori@med.nagoya-u.ac.jp (T.M.); moto@med.nagoya-u.ac.jp (M.N.); 2 Department of Rehabilitation, Toyota Memorial Hospital, Toyota 471-8513, Japan; jabbathehutt626@gmail.com; 3 Department of Orthopaedic Surgery, Nagoya University Graduate School of Medicine, Nagoya 466-8550, Japan; automaticeye.0213@gmail.com (H.I.); ysk0568@yahoo.co.jp (Y.O.); gamitake1919@gmail.com (Y.T.)

**Keywords:** sarcopenic severity, frailty, physical performance, total hip replacement, hip surgical procedures, postoperative outcomes

## Abstract

The outcome of total hip arthroplasty (THA) in patients with end-stage arthritis of the hip is associated with preoperative physical status. This study was performed to examine the relationship between the preoperative severity of sarcopenia and clinical outcomes after THA. This retrospective cohort study was performed among 306 consecutive patients (mean age: 63.7 ± 12.9 years, 222 women) undergoing THA at a university hospital. The severity of sarcopenia was determined based on the skeletal muscle mass index (SMI), handgrip strength, and gait speed according to the criteria of the Asian Working Group for Sarcopenia 2019. The severe sarcopenia prevalence rate was 10.6%. Severe sarcopenia was significantly associated with the risk of delayed functional recovery (adjusted odds ratio, 2.82; 95% confidence interval, 1.03–7.72; *p* = 0.043) compared with the non-sarcopenia group after adjusting for pre-existing risk factors, including preoperative hip function and physical activity. The addition of SMI, handgrip strength, and gait speed to the model for risk of functional recovery delay significantly increased the area under the receiver operating characteristic curve (*p* = 0.038). Severe sarcopenia was significantly associated with poorer hip function and patient-reported outcomes at 6 months after surgery compared with the non-sarcopenia group. Severe sarcopenia was adversely associated with postoperative clinical outcomes in patients undergoing THA.

## 1. Introduction

Total hip arthroplasty (THA) is commonly performed for the treatment of end-stage osteoarthritis of the hip [1], a leading cause of disability affecting an estimated 40 million people worldwide [2,3]. As persistent pain and functional decline are major indicators of THA, patients frequently have limited physical function and mobility prior to surgery. THA has been shown to provide pain relief, mobility enhancement, and restoration of function. It has been reported that the postoperative clinical outcomes of THA, including patient satisfaction, can be predicted by preoperative physical status [4]. Studies of the risk factors associated with the adverse outcomes of THA are required to identify possible interventions that may lead to improvements in patient satisfaction and functional recovery.

Sarcopenia is a progressive generalized skeletal muscle disorder associated with accelerated loss of muscle mass and strength, the incidence of which increases with advanced age but can also occur in younger patients in the context of chronic disease [5]. Sarcopenia is associated with adverse outcomes, including falls, frailty, and disability, with poor prognosis and reduced quality of life (QoL) [5]. In addition, preoperative sarcopenia was reported to be associated with increased postoperative complications and mortality as well as greater medical costs in patients undergoing THA [6,7]. Although sarcopenia has mostly been defined based on reduced muscle mass and/or strength, the current guidelines include an assessment of physical performance for determination of its severity [5,8]. Severe sarcopenia with markedly reduced muscle mass, strength, and physical performance is associated with poor cognitive function, greater risk of falls, and increased mortality risk in community-dwelling adults [9,10,11]. However, there have been no previous reports regarding the association between severe sarcopenia and post-THA outcomes.

We postulated that patients with severe sarcopenia would have poorer clinical out-comes than those without sarcopenia and examined the association between preoperative sarcopenia severity and clinical outcomes following THA.

## 2. Materials and Methods

### 2.1. Study Population

This retrospective cohort study was performed among all consecutive patients receiving rehabilitation following THA at Nagoya University Hospital between June 2016 and June 2020. Patients without a preoperative assessment of sarcopenic status were excluded. A standard rehabilitation program consisting of progressively improving walking ability, other functional activities, and walking stairs was begun on postoperative day (POD) 1. Patients also participated in a progressive program involving exercises to improve range of motion (ROM), muscle strength, and function. This study was approved by the Institutional Review Board of Nagoya University Hospital and performed in accordance with the tenets of the Declaration of Helsinki and the Japanese Ethical Guidelines for Medical and Health Research Involving Human Subjects. Information regarding this study, including the objectives and inclusion and exclusion criteria, was published on a website, and the participants were free to opt out of this study at any time.

### 2.2. Data Collection

Details regarding clinical presentation and demographic and biochemical information for all patients were obtained from their electronic medical records. Hip function was classified preoperatively and at a 6-month follow-up based on the Japanese Orthopaedic Association (JOA) hip score, which allocates 40 points for pain, 20 points for ROM, 20 points for walking ability, and 20 points for activities of daily living (ADL) with a maximum total score of 100 points, where 100 indicates best performance [12,13]. The University of California Los Angeles (UCLA) activity score, with scores ranging from 1 (very low) to 10 (very high), was used to assess preoperative physical activity [14,15]. Orthopedic surgeons performed postoperative follow-ups by routine physical examination, pelvic radiography, and laboratory blood analyses.

### 2.3. Evaluation of Sarcopenia

Appendicular skeletal muscle mass, handgrip strength, and gait speed were measured by trained physiotherapists to determine the severity of sarcopenia. Sarcopenia was defined as low muscle mass and strength or low physical performance, while severe sarcopenia was characterized by the presence of all three conditions in accordance with the Asian Working Group for Sarcopenia 2019 (AWGS2019) [8].

The skeletal muscle mass index (SMI) was calculated by dividing the appendicular skeletal muscle mass measured by bioelectrical impedance analysis (InBody 720; InBody Inc., Tokyo, Japan) by the square of height (m^2^). Low appendicular skeletal muscle mass was defined as SMI < 7.0 kg/m^2^ for men and <5.7 kg/m^2^ for women [8]. Muscle strength was evaluated by measuring handgrip strength using a digital dynamometer (TKK 5101 Grip-D; Takei, Tokyo, Japan) with the patient in the standing position. Each patient performed two maximal isometric voluntary contractions of both hands for 3 s each, and the greatest strength expressed as an absolute value (in kg) was used in the analyses. Low muscle strength was defined as handgrip strength <28 kg for men and <18 kg for women [8]. Physical performance was evaluated based on the usual gait speed measured over the middle 10 m of a 16 m walkway with the use of any required assistive devices. Low physical performance was defined as gait speed <1.0 m/s [8].

### 2.4. Computed Tomography, Physical Function, and Mental Health Evaluations

Thigh muscle area, subcutaneous fat area, and circumference were determined semi-automatically (SYNAPSE VINCENT™; Fujifilm Medical Co., Ltd., Tokyo, Japan) on preoperative pelvic computed tomography (CT) scans obtained within 1 month before surgery at the mid-thigh level between the anterior superior iliac crest and the patella [16]. Muscle and fat areas were quantified based on thresholds of −29 to +150 and −190 to −30 Hounsfield units, respectively. Image analysis was performed in a blinded manner with regard to clinical information to minimize bias in measurements and calculations.

Calf circumference was measured to the nearest 1 mm at the point of the greatest circumference using a plastic tape measure with the patient in the supine position. Leg strength was evaluated by measuring maximal hip abductor strength and knee extensor strength using a handheld dynamometer (m-Tas; ANIMA, Tokyo, Japan). Hip abductor strength was assessed with the patient in the supine position with neutral hip adduction/abduction. Knee extensor strength was measured with the patient in the sitting position with the knee joint fixed at 60°. Maximal isometric voluntary contractions for 5 s each time were determined twice successively for both legs, and the length (m) of the lever arm was measured from the estimated joint center of rotation to the center of the dynamometer. The greatest values of strength on the right and left sides were used for the analyses, and the dynamometer variable (kgf) and lever arm length (m) were multiplied to obtain torque (kgf*m). ADL was determined based on the Barthel Index, routinely recorded in nursing and rehabilitation summaries, with scores ranging from 0 to 100, where 100 indicates independent in basic ADL.

Anxiety and depression were measured using the 14-item Hospital Anxiety and Depression Scale (HADS) [17], which has seven items for each of anxiety and depression scored from 0 to 3 with subscale scores ranging from 0 to 21, where higher scores indicate greater anxiety or depression. Patient-reported outcomes were evaluated preoperatively and at a 6-month follow-up after surgery using the Japanese Orthopaedic Association Hip-Disease Evaluation Questionnaire (JHEQ) [18], a validated self-administered questionnaire to determine the QoL of Asian patients with hip disease consisting of items on pain (0–28 points), function (0–28 points), and mental (0–28 points), with a total score ranging from 0 (worst) to 84 (best) [19]. Patient satisfaction with a hip condition was evaluated using a visual analog scale (VAS) ranging from 0 mm (greatest satisfaction) to 100 mm (greatest dissatisfaction).

### 2.5. Clinical Outcomes

Functional recovery, defined as the ability to walk over a distance of 100 m regardless of the use of a walking aid, was evaluated daily, and the primary end point of this study was functional recovery on POD7. The secondary end points of this study were the Barthel Index at hospital discharge, the incidence of hospital-acquired disability, length of hospital stay, non-home discharge, and the occurrence of adverse events within 6 months after surgery. A decrease in the Barthel Index of at least 5 points on the day before hospital discharge compared with admission was taken to indicate hospital-acquired disability [20]. Adverse events included wound complications, infection, dislocation, loosening, periprosthetic fracture, reoperation, and all-cause unplanned readmission and mortality.

### 2.6. Statistical Analysis

Continuous variables with a normal distribution are shown as the mean ± standard deviation, while those with a non-normal distribution are shown as the median and interquartile range. Categorical variables are expressed as numbers and percentages. Differences between the three groups classified by sarcopenia status (i.e., the non-sarcopenia group, sarcopenia group, and severe sarcopenia group) were evaluated by one-way analysis of variance or the Kruskal–Wallis test for continuous variables and the chi-squared or Fisher’s exact test for dichotomous variables, as appropriate. Differences between the severe sarcopenia and no sarcopenia assessment groups were evaluated by the unpaired Student’s *t* test, Mann–Whitney U test, chi-squared test, or Fisher’s exact test as appropriate.

Functional recovery delay on POD7 and non-home discharge risks were examined by logistic regression analysis of sarcopenia status with adjustment for age, sex, body mass index (BMI), JOA hip score, living alone, and UCLA activity score as potential confounders based on their clinical importance and the results of previous studies. Adjusted odds ratios (ORs) are reported with the corresponding 95% confidence intervals (CIs). Receiver operating characteristic (ROC) curve analysis of the study end points, i.e., functional recovery delay on POD7 and non-home discharge, was performed to compare the incremental prognostic values of adding assessments of sarcopenia to the baseline model incorporating risk factors, including all variables used for adjustment (i.e., age, sex, BMI, JOA hip score, living alone, and UCLA activity score), and the areas under the curves (AUCs) were compared according to the method of DeLong et al. [21]. Decision curve analysis was performed to quantify the net benefits at different threshold probabilities to assess the clinical usefulness of the model [22].

In all analyses, two-tailed *p* < 0.05 was taken to indicate statistical significance, and the Bonferroni correction was applied for multiple comparisons. Statistical analyses were performed using R version 3.2.1 (R Foundation for Statistical Computing, Vienna, Austria).

## 3. Results

After excluding 32 of the 338 THA patients treated during the study period because of a lack of preoperative assessment, a total of 306 patients including 52 for whom sarcopenia assessment could not be completed because of severe physical function decline or pain (no sarcopenia assessment group; *n* = 52) were included in this study. The study population consisted of 222 women (72.5%) and 84 men (27.5%) with a mean age of 63.7 ± 12.9 years. Of the study population, 78.1% had osteoarthritis and 17.3% were undergoing revision surgeries. 

**Figure 1:**
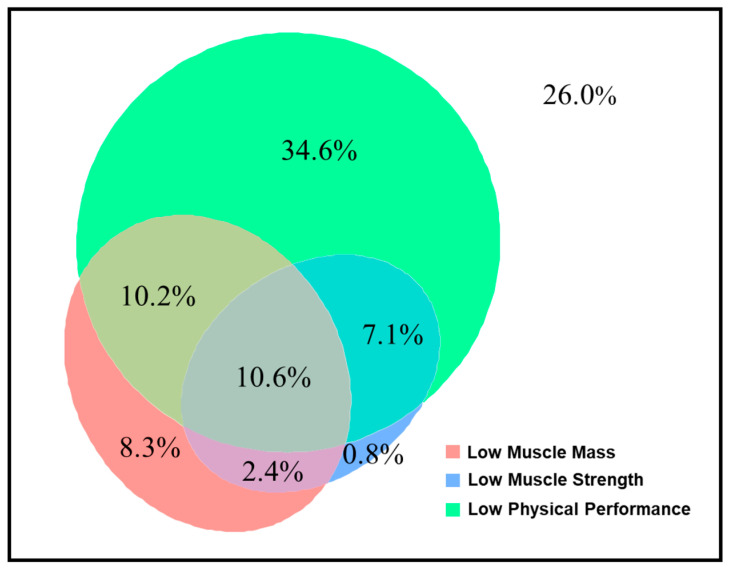
Proportions of overlap and non-overlap among sarcopenia domains.

**Figure A1:**
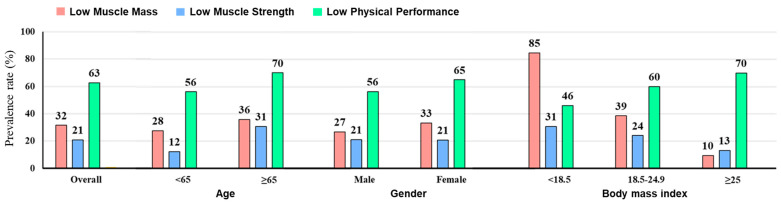
Prevalence rates of sarcopenia domains.

The patients for whom preoperative sarcopenia assessments were available (*n* = 254) were divided into the non-sarcopenia group (*n* = 195, 76.8%), sarcopenia group (*n* = 32, 12.6%), and severe sarcopenia group (*n* = 27, 10.6%). These patients had a mean SMI of 7.4 ± 1.0 cm^2^/m^2^ for men and 6.1 ± 1.0 cm^2^/m^2^ for women, a mean handgrip strength of 35.2 ± 8.4 kg for men and 22.2 ± 5.7 kg for women, and a mean usual gait speed of 0.83 ± 0.38 m/s. Low muscle mass, low muscle strength, and low physical performance had prevalence rates of 32%, 21%, and 63%, respectively, with the highest percentages of low physical performance across various subgroups except in patients with BMI < 18.5 kg/m^2^ (Figure A1). Euler diagrams demonstrated a significant overlap in the assessments of sarcopenia (Figure 1).

**Figure A2:**
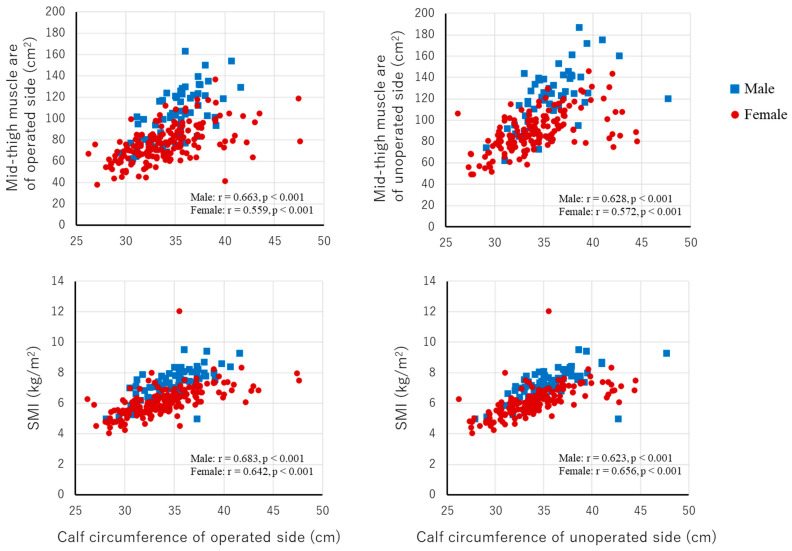
Associations between calf circumference and mid-thigh muscle area and SMI. SMI, skeletal muscle index.

Table 1 presents the baseline characteristics of the total study population and each subgroup classified by preoperative sarcopenia status. Compared with the non-sarcopenia group, the severe sarcopenia group had significantly greater age, lower BMI, lower prevalence of living alone, lower UCLA activity score, and lower hemoglobin levels. There were no significant differences in sex, histological type, preoperative hip function, pain, comorbidities, smoking status, or surgery-related factors between the groups. The JOA hip score, UCLA activity score, and albumin level were significantly lower in the no sarcopenia assessment group than in the severe sarcopenia group (Table A1). Mid-thigh muscle area, limb circumferences, muscle strength, peak expiratory flow, and JHEQ function score were significantly lower in the severe sarcopenia group than in the non-sarcopenia group (Table 2). HADS anxiety and depression scores and the JHEQ mental score were significantly lower in the no sarcopenia assessment group than in the severe sarcopenia group (Table A2). Calf circumference was significantly correlated with mid-thigh muscle area assessed by CT and SMI in the total patient population (Figure A2).

**Figure 2:**
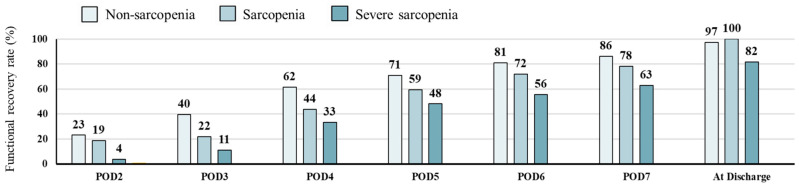
Proportions of patients showing functional recovery after surgery.

A total of 210 patients achieved functional recovery on POD7 (82.7%), and 180 were discharged home (70.9%). The severe sarcopenia group had consistently lower daily functional recovery rates than the non-sarcopenia group (63% and 86% on POD7) (Figure 2). 

The risks of functional recovery delay (adjusted OR, 2.82; 95% CI, 1.03–7.72; *p* = 0.043) and non-home discharge (adjusted OR, 2.72; 95% CI, 1.01–7.31; *p* = 0.047) were higher in the severe sarcopenia group than in the non-sarcopenia group based on the logistic regression analysis after adjusting for possible confounding factors (Table 3).

**Figure 3:**
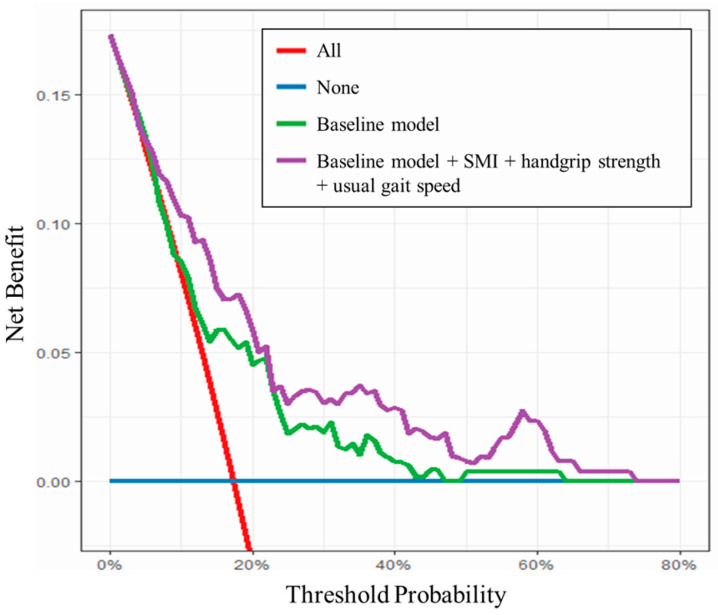
Decision curve analysis for functional recovery delay.

The baseline logistic regression model and further models with the addition of sarcopenia assessment on ROC curve analysis are shown in Table 4. In the model with the addition of all sarcopenia assessments to the baseline model, the AUC for functional recovery delay on POD7 was significantly higher than in the baseline model (0.770; 95% CI 0.697–0.845; *p* = 0.038). Although the difference was not significant, the model with the addition of all sarcopenia assessments to the baseline model showed the greatest AUC for non-home discharge (0.753; 95% CI, 0.688–0.818; *p* = 0.371). However, there was a significant increase in AUC for non-home discharge with the inclusion of the number of days until functional recovery in the baseline model (0.807; 95% CI, 0.748–0.866; *p* < 0.001). The baseline model with the addition of all sarcopenia assessments showed a better predictive ability for functional recovery delay than the baseline model between threshold probabilities of 10% and 60% (Figure 3).

The postoperative physical function and clinical outcomes are shown in Table A3. Muscle strength, physical function, and Barthel Index at discharge were significantly lower and hospital stays were significantly longer in the severe sarcopenia group than in the non-sarcopenia group. However, there were no significant differences in pain or changes in these parameters between the groups. Postoperative clinical outcomes, including functional recovery delay and non-home discharge, were not significantly different between the severe sarcopenia group and no sarcopenia assessment group (Table A4).

The clinical outcomes at 6 months after surgery are shown in Table 5. The JOA hip score and JHEQ total score were significantly lower, while the JHEQ VAS dissatisfaction scale score was significantly higher, in the severe sarcopenia group than in the non-sarcopenia group. However, the incidence of adverse events within 6 months after surgery was not significantly different between these two groups. Clinical outcomes at 6 months after surgery were not significantly different between the severe sarcopenia group and no sarcopenia assessment group (Table A5).

## 4. Discussion

This study was performed to investigate the association between preoperative sarcopenic status and clinical outcomes following THA. Approximately 25% of patients in the present study had preoperative sarcopenia. The rate of severe sarcopenia was 10.6%, which was associated with increased incidence rates of functional recovery delay and non-home discharge, lower ADL level at discharge, and longer hospital stay in comparison with the non-sarcopenia group. SMI, handgrip strength, and usual gait speed as indicators of sarcopenia assessed in the present study had complementary predictive capability to known risk factors, such as preoperative hip function and physical activity, for risk of functional recovery delay after THA. Hip function and patient-reported QoL outcomes, including patient satisfaction, were significantly poorer in the severe sarcopenia group than in the non-sarcopenia group at 6 months after THA. These observations indicate the need for comprehensive evaluation taking preoperative sarcopenia status into consideration in THA.

Consistent with previous reports, our study population had a preoperative sarcopenia prevalence rate of 23.2%, which was reported to be associated with unfavorable short- and long-term clinical outcomes following THA [7,23]. In these previous studies, however, sarcopenia was evaluated based only on muscle mass and handgrip strength, while we defined sarcopenia using the criteria of the AWGS2019, involving a comprehensive assessment of muscle mass, muscle strength (monitored by handgrip strength), and physical performance (monitored by gait speed) [8]. Walking ability is limited by diseases of the hip, and preoperative gait speed was reported previously to be a good prognostic indicator for functional recovery after THA [24]. Measures of physical performance are recommended for assessment of the severity of sarcopenia [5,8]. The highest prevalence rate of low physical performance was 63% in the present study, and severe sarcopenia, but not sarcopenia, was related to poor outcomes, including functional recovery delay in the short term, poor hip function, and patient-reported outcomes at 6 months after surgery. The results presented here indicated the benefit of examining the severity of sarcopenia preoperatively in patients prior to THA. Previous large observational studies showed the association between frailty, which is closely related to sarcopenia, and increased rates of complications and mortality after THA [25,26], although the present study showed no significant differences in rates of adverse events within 6 months postoperatively according to sarcopenia status. As the statistical power of the present study may have been too low to detect such associations because of the small sample size and limited number of recorded events, further studies are required in larger numbers of patients undergoing THA. The gait speed of patients with specific diseases, such as hip fracture, cannot be assessed before surgery, as they are unable to walk. Preoperative mental status and ADL level were poorer in our no sarcopenia assessment group compared with the severe sarcopenia group, and these groups had no significant differences in postoperative clinical outcomes. The results presented here suggested that greater attention may be required in the management of patients in whom sarcopenia cannot be assessed in addition to those with severe sarcopenia.

Even in the logistic regression model adjusted for potential confounding factors, severe sarcopenia was shown to be a significant independent predictor of functional recovery delay and non-home discharge after THA. The model with the addition of all sarcopenia assessments to the previously identified risk factors, but not any individual factor, had the highest AUC for functional recovery delay (AUC, 0.770), suggesting that a multi-item composite measure would outperform the assessment of individual items in patients undergoing THA. The ability to predict functional recovery delay regardless of risk was consistently increased by the addition of sarcopenia assessments to previously identified risk factors (threshold probabilities, 10–60%). To our knowledge, this is the first report of the additional predictive capability of preoperative sarcopenia assessments consisting of multiple objectively measured items in THA patients. There was no improvement in the AUC for non-home discharge by the addition of sarcopenia assessments to the baseline model in the present study. The significant increase in AUC for non-home discharge by the inclusion of the number of days to functional recovery in the baseline model (AUC, 0.807) suggested the importance of postoperative rehabilitation for improvement of the postoperative clinical course in patients undergoing THA.

The results of the present study have implications for both clinical practice and the design of future clinical studies regarding THA. For medical treatment planning and to improve clinical outcomes in vulnerable populations, it is necessary to develop means of accurately stratifying patient risk. The accurate assessment of preoperative sarcopenia is necessary because suitable interventions, including nutritional recommendations and rehabilitation, can be implemented both pre- and postoperatively [27,28,29]. Exercise represents a nonpharmacological intervention that can improve the pain, physical function, QoL, and mental health of patients with hip osteoarthritis [30]. Resistance training and the use of nutritional supplements, including branched-chain amino acids, vitamin D, whey protein, and hydroxymethylbutyrate-enriched milk, can play important roles in ameliorating sarcopenia [8], which may improve the outcomes of frail patients following THA. However, the effects of these interventions on severe sarcopenia have yet to be determined, and whether such amelioration of sarcopenia can improve clinical outcomes remains to be determined. Further studies are therefore required to facilitate clinical decision-making processes in patients requiring THA.

This study had several limitations. First, this was a single-center observational study in a small population of Asian patients, and the small number of events may have increased the risk of type I errors. Second, substantially longer hospital stays in Japan compared with Western countries may have affected the results of this study, especially with regard to non-home discharge. Further studies in larger cohorts and other populations are required to validate the prognostic effects of preoperative sarcopenic status assessment. Third, the retrospective nature of this study meant that the accuracy of some variables was dependent on the quality of the medical records. Finally, although multivariate analysis may mitigate bias after adjustment for previously identified risk factors, other factors that were not measured or for which the models were not adjusted, such as cognitive function and changes in baseline variables, can also result in residual bias. Further studies are therefore required to confirm the findings presented here.

## 5. Conclusions

This study showed that preoperative severe sarcopenia was associated with the risks of functional recovery delay and non-home discharge in patients undergoing THA, and this condition adversely affected hip function and patient-reported outcomes, including patient satisfaction with hip condition, at 6 months postoperatively. This study suggested the beneficial effects of determining preoperative sarcopenia status for accurate risk stratification in patients requiring THA.

## Figures and Tables

**Table 1:** Baseline patient characteristics.

		Overall (n = 254)			Non-Sarcopenia			Sarcopenia			Severe Sarcopenia			*p* Value
					(n = 195; 76.8%)			(n = 32; 12.6%)			(n = 27; 10.6%)			
Age (years)		62.5	±	12.2	61.5	±	11.9	61.7	±	12.9	70.8	±	10.6 ^a,b^	0.001
	≥65	117 (46.1)			80 (41.0)			17 (53.1)			20 (74.1)			0.004
Male		71 (28.0)			60 (30.8)			4 (12.5)			7 (25.9)			0.099
BMI (kg/m^2^)		24.0	±	4.0	24.8	±	4.1	21.2	±	2.6 ^a^	22.1	±	2.8 ^a^	<0.001
BMI group														<0.001
	<18.5	13 (5.1)			6 (3.1)			5 (15.6)			2 (7.4)			
	18.5–24.9	158 (62.2)			111 (56.9)			25 (78.1)			22 (81.5)			
	≥25	83 (32.7)			78 (40.0)			2 (6.2)			3 (11.1)			
Etiology														0.114
	Osteoarthritis	203 (79.9)			162 (83.1)			23 (71.9)			18 (66.7)			
	Osteonecrosis of the femoral head	36 (14.2)			24 (12.3)			7 (21.9)			5 (18.5)			
	Other	15 (5.9)			9 (4.6)			2 (6.2)			4 (14.8)			
JOA hip score (points)		59	±	15	60	±	15	60	±	11	56	±	18	0.420
Hip flexion ROM (°)														
	Operated side	84	±	23	85	±	22	82	±	26	75	±	22	0.083
	Contralateral side	101	±	24	103	±	22	97	±	29	93	±	24	0.061
VAS for pain (mm)														
	Average	31 [16, 55]			30 [15, 55]			34 [18, 52]			42 [20, 55]			0.783
	Maximum	60 [30, 79]			62 [30, 81]			57 [25, 74]			53 [35, 79]			0.530
Living alone		44 (17.3)			39 (20.0)			5 (15.6)			0 (0.0) ^a^			0.035
Diabetes		40 (15.7)			35 (17.9)			1 (3.1)			4 (14.8)			0.102
Charlson comorbidity index		0.67	±	1.03	0.61	±	1.00	0.88	±	1.29	0.89	±	0.85	0.205
Never a smoker		187 (73.6)			138 (70.8)			26 (81.2)			23 (85.2)			0.162
UCLA activity score		4.5	±	1.6	4.7	±	1.6	4.5	±	1.5	3.6	±	1.4 ^a^	0.004
Laboratory findings														
	Albumin (g/dL)	4.1	±	0.3	4.2	±	0.4	4.2	±	0.3	4.0	±	0.3	0.060
	Hemoglobin (g/dL)	13.1	±	1.5	13.3	±	1.5	12.5	±	1.0 ^a^	12.4	±	1.3 ^a^	<0.001
	Creatinine (mg/dL)	0.8	±	0.4	0.8	±	0.4	0.6	±	0.2	0.7	±	0.3	0.097
	eGFR (mL/min/1.73 m^2^)	73.5	±	18.4	71.9	±	17.5	82.3	±	18.2 ^a^	74.2	±	22.0	0.012
	CRP (mg/L)	0.08 [0.05, 0.17]			0.08 [0.05, 0.18]			0.07 [0.04, 0.15]			0.11 [0.08, 0.18]			0.187
SMI (kg/m^2^)														
	Male	7.4	±	1.0	7.6	±	0.8	5.8	±	0.7 ^a^	6.3	±	0.6 ^a^	<0.001
	Female	6.1	±	1.0	6.5	±	0.8	5.2	±	0.4 ^a^	5.1	±	0.4 ^a^	<0.001
Handgrip strength (kg)														
	Male	35.2	±	8.4	37.0	±	7.7	26.3	±	4.0 ^a^	24.6	±	3.5 ^a^	<0.001
	Female	22.2	±	5.7	23.7	±	5.1	21.0	±	2.8 ^a^	13.4	±	3.8 ^a^	<0.001
Usual gait speed (m/s)		0.83	±	0.38	0.88	±	0.39	0.70	±	0.32 ^a^	0.62	±	0.26 ^a^	<0.001
Type of surgery														0.916
	Primary	214 (84.3)			165 (84.6)			27 (84.4)			22 (81.5)			
	Revision	40 (15.7)			30 (15.4)			25 (15.6)			5 (18.5)			
Operation time (min)		118 [92, 165]			118 [94, 163]			118 [84, 164]			122 [80, 186]			0.808
Blood loss during surgery (g)		472 [303, 713]			472 [318, 720]			519 [268, 700]			416 [195, 674]			0.550

BMI, body mass index; CRP, C-reactive protein; eGFR, estimated glomerular filtration rate; JOA, Japanese Orthopaedic Association; ROM, range of motion; SMI, skeletal muscle mass index; UCLA, University of California Los Angeles; VAS, visual analog scale. ^a^ Significant difference from the non-sarcopenia group. ^b^ Significant difference from the sarcopenia group.

**Table 2:** Associations between preoperative sarcopenic status and physical and mental status.

		Overall (n = 254)			Non-Sarcopenia			Sarcopenia			Severe Sarcopenia			*p* Value
					(n = 195; 76.8%)			(n = 32; 12.6%)			(n = 27; 10.6%)			
Mid-thigh muscle area (cm^2^)														
	Operated side	84.6	±	22.9	90.1	±	22.3	65.5	±	13.1 ^a^	66.6	±	13.0 ^a^	<0.001
	Contralateral side	98.4	±	25.3	105.3	±	23.8	75.2	±	12.1 ^a^	77.8	±	19.3 ^a^	<0.001
Mid-thigh muscle attenuation (HU)														
	Operated side	42.4	±	8.3	42.8	±	8.3	43.1	±	8.5	39.1	±	8.2	0.099
	Contralateral side	46.7	±	7.6	47.0	±	7.5	47.7	±	7.4	44.1	±	8.0	0.149
Mid-thigh subcutaneous fat area (cm^2^)														
	Operated side	64.4	±	34.1	67.2	±	36.7	58.7	±	19.0	50.4	±	23.1	0.037
	Contralateral side	57.0	±	29.7	59.3	±	32.2	53.4	±	17.7	45.7	±	18.6	0.071
Mid-thigh circumference (cm)														
	Operated side	41.4	±	5.2	42.6	±	4.6	38.1	±	3.2 ^a^	36.6	±	6.7 ^a^	<0.001
	Contralateral side	42.6	±	4.7	43.9	±	4.4	38.7	±	3.0 ^a^	38.6	±	3.3 ^a^	<0.001
Calf circumference (cm)														
	Operated side	34.2	±	3.5	35.2	±	3.3	31.0	±	2.0 ^a^	31.5	±	2.0 ^a^	<0.001
	Contralateral side	34.5	±	3.6	35.6	±	3.4	31.1	±	2.0 ^a^	31.7	±	2.2 ^a^	<0.001
Hip abductor strength (kgf·m)														
	Operated side	2.8	±	2.0	3.1	±	2.0	2.3	±	1.7	1.8	±	1.3 ^a^	0.002
	Contralateral side	2.7	±	1.5	3.0	±	1.6	2.0	±	0.9 ^a^	1.9	±	0.9 ^a^	<0.001
Knee extensor strength (kgf·m)														
	Operated side	4.6	±	2.5	5.1	±	2.6	3.4	±	1.7 ^a^	2.8	±	1.4 ^a^	<0.001
	Contralateral side	5.5	±	2.9	6.0	±	3.0	3.7	±	1.3 ^a^	3.6	±	1.7 ^a^	<0.001
Maximal gait speed (m/s)		1.20	±	0.40	1.30	±	0.40	1.10	±	0.30 ^a^	0.90	±	0.30 ^a^	<0.001
Pulmonary function														
	VC (%predicted)	105.6	±	16.1	106.6	±	15.7	102.0	±	17.5	102.2	±	17.4	0.175
	FEV_1_ (%predicted)	100.9	±	19.7	102.4	±	18.5	96.6	±	26.3	95.1	±	17.3	0.080
	FEV_1_/FVC	108.8	±	18.4	110.2	±	17.0	102.8	±	24.0	105.9	±	19.5	0.072
	PEF (%predicted)	104.7	±	21.7	107.5	±	21.9	100.4	±	18.6	89.6	±	16.4 ^a^	<0.001
Barthel Index (points)		100 [100, 100]			100 [100, 100]			100 [100, 100]			100 [90, 100]			<0.001
HADS														
	Anxiety	6	±	5	6	±	5	6	±	4	5	±	3	0.589
	Depression	6	±	4	6	±	4	6	±	3	5	±	4	0.538
JHEQ score (points)														
	Total	29	±	16	29	±	17	28	±	13	26	±	12	0.656
	Pain	11	±	7	11	±	7	10	±	7	10	±	8	0.784
	Function	6	±	6	7	±	6	6	±	4	4	±	4 ^a^	0.039
	Mental	12	±	7	11	±	7	12	±	6	12	±	5	0.761
JHEQ VAS dissatisfaction scale (mm)		76	±	28	77	±	26	69	±	32	75	±	31	0.306

FEV_1_, forced expiratory volume in 1 s; FVC, forced vital capacity; HADS, hospital anxiety and depression scale; HU, Hounsfield units; JHEQ, Japanese Orthopaedic Association Hip-Disease Evaluation Questionnaire; PEF, peak expiratory flow; VC, vital capacity; VAS, visual analog scale. ^a^ Significant difference from the non-sarcopenia group.

**Table 3:** Logistic regression analyses for postoperative clinical outcomes.

			Univariate Analyses			Multivariate Analyses ^a,b^		
Variable		Event No.	OR	95% CI	*p* Value	OR	95% CI	*p* Value
Functional recovery delay on POD7								
	Non-sarcopenia	27 (13.8)	1.00	[Reference]		1.00	[Reference]	
	Sarcopenia	7 (21.9)	1.74	0.69–4.42	0.243	2.14	0.75–6.08	0.153
	Severe sarcopenia	10 (37.0)	3.66	1.52–8.83	0.004	2.82	1.03–7.72	0.043
Non-home discharge								
	Non-sarcopenia	50 (25.6)	1.00	[Reference]		1.00	[Reference]	
	Sarcopenia	10 (31.2)	1.32	0.58–2.97	0.506	1.95	0.75–5.06	0.169
	Severe sarcopenia	14 (51.9)	3.12	1.37–7.09	0.007	2.72	1.01–7.31	0.047

CI, confidence interval; OR, odds ratio; POD, postoperative day. ^a^ Multivariate analyses for functional recovery delay, adjusted for age, sex, BMI, JOA hip score, and UCLA activity score. ^b^ Multivariate analyses for non-home discharge, adjusted for age, sex, BMI, JOA hip score, living alone, and UCLA activity score.

**Table 4:** Predictive value analysis for postoperative outcomes.

Model		AUC	95% CI			*p* Value
Functional recovery delay on POD7						
	Baseline model ^a^	0.694	0.604	-	0.784	[Reference]
	Baseline model + SMI	0.722	0.634	-	0.810	0.247
	Baseline model + handgrip strength	0.746	0.662	-	0.831	0.090
	Baseline model + usual gait speed	0.743	0.666	-	0.820	0.086
	Baseline model + SMI + handgrip strength + usual gait speed	0.770	0.694	-	0.845	0.038
Non-home discharge						
	Baseline model ^b^	0.739	0.673	-	0.804	[Reference]
	Baseline model + SMI	0.749	0.684	-	0.813	0.414
	Baseline model + handgrip strength	0.748	0.683	-	0.813	0.515
	Baseline model + usual gait speed	0.739	0.673	-	0.805	1.000
	Baseline model + SMI + handgrip strength + usual gait speed	0.753	0.688	-	0.818	0.371
	Baseline model + days to functional recovery	0.807	0.748	-	0.866	<0.001

AUC, area under the curve; CI, confidence interval; POD, postoperative day; SMI, skeletal muscle index. ^a^ Baseline model: age, sex, body mass index, JOA hip score, and UCLA activity score. ^b^ Baseline model: age, sex, body mass index, JOA hip score, living alone, and UCLA activity score.

**Table 5:** Clinical outcomes at 6 months after surgery.

		Overall (n = 254)			Non-Sarcopenia			Sarcopenia			Severe Sarcopenia			*p* Value
					(n = 195; 76.8%)			(n = 32; 12.6%)			(n = 27; 10.6%)			
JOA hip score (points) (n = 241)		84	±	14	86	±	13	81	±	14	76	±	17 ^a^	0.001
JHEQ score (points) (n = 200)														
	Total	55	±	17	57	±	17	49	±	17	50	±	17	0.037
	Pain	24	±	6	24	±	6	22	±	6	23	±	7	0.199
	Function	13	±	8	14	±	8	10	±	8	8	±	7 ^a^	0.003
	Mental	19	±	7	19	±	7	17	±	6	19	±	7	0.478
JHEQ VAS dissatisfaction scale (mm)		21	±	27	19	±	26	23	±	25	35	±	38 ^a^	0.044
Adverse events within 6 months after surgery		36 (14.2)			29 (14.9)			3 (9.4)			4 (14.8)			0.707

JOA, Japanese Orthopaedic Association; JHEQ, Japanese Orthopaedic Association Hip-Disease Evaluation Questionnaire; VAS, visual analog scale. ^a^ Significant difference from the non-sarcopenia group.

## Data Availability

The data that support the findings of this study are available from the corresponding author upon reasonable request. The data are not publicly available because of privacy restrictions.

## Appendix B

**Table A1:** Baseline patient characteristics between two groups.

		Severe Sarcopenia			No Sarcopenia Assessment			*p* Value
		(n = 27)			(n = 52)			
Age (years)		70.8	±	10.6	69.7	±	14.6	0.720
	≥65	20 (74.1)			33 (63.5)			0.451
Male		7 (25.9)			13 (25.0)			1.000
BMI (kg/m^2^)		22.1	±	2.8	23.4	±	4.3	0.166
BMI group								0.073
	<18.5	2 (7.4)			7 (13.5)			
	18.5–24.9	22 (81.5)			29 (55.8)			
	≥25	3 (11.1)			16 (30.8)			
Etiology								0.316
	Osteoarthritis	18 (66.7)			36 (69.2)			
	Osteonecrosis of the femoral head	5 (18.5)			4 (7.7)			
	Other	4 (14.8)			12 (23.1)			
JOA hip score (points)		56	±	18	46	±	19	0.034
Hip flexion ROM (°)								
	Operated side	75	±	22	68	±	26	0.273
	Contralateral side	93	±	24	90	±	33	0.783
VAS for pain (mm)								
	Average	42 [20, 55]			45 [24, 65]			0.526
	Maximum	53 [35, 79]			64 [30, 86]			0.261
Living alone		0 (0.0)			12 (23.1)			0.006
Diabetes		4 (14.8)			8 (15.4)			1.000
Charlson comorbidity index		0.89	±	0.85	0.89	±	1.29	0.957
Never a smoker		23 (85.2)			39 (75.0)			0.392
UCLA activity score		3.6	±	1.4	2.8	±	1.4	0.017
Laboratory findings								
	Albumin (g/dL)	4.0	±	0.3	3.7	±	0.6	0.023
	Hemoglobin (g/dL)	12.4	±	1.3	12.1	±	1.6	0.309
	Creatinine (mg/dL)	0.7	±	0.3	1.0	±	1.2	0.257
	eGFR (mL/min/1.73 m^2^)	74.2	±	22.0	74.7	±	34.4	0.947
	CRP (mg/L)	0.11 [0.08, 0.18]			0.22 [0.07, 0.75]			0.091
Type of surgery								0.583
	Primary	22 (81.5)			39 (75.0)			
	Revision	5 (18.5)			13 (25.0)			
Operation time (min)		122 [80, 186]			146 [113, 206]			0.075
Blood loss during surgery (g)		416 [195, 674]			501 [279, 206]			0.075

BMI, body mass index; CRP, C-reactive protein; eGFR, estimated glomerular filtration rate; JOA, Japanese Orthopaedic Association; ROM, range of motion; UCLA, University of California Los Angeles; VAS, visual analog scale.

**Table A2:** Associations between preoperative sarcopenic status and physical and mental status between two groups.

		Severe Sarcopenia			No Sarcopenia Assessment			*p* Value
		(n = 27)			(n = 52)			
Mid-thigh muscle area (cm^2^)								
	Operated side	66.6	±	13.0	73.0	±	21.6	0.170
	Contralateral side	77.8	±	19.3	82.2	±	23.2	0.417
Mid-thigh muscle attenuation (HU)								
	Operated side	39.1	±	8.2	35.2	±	8.2	0.051
	Contralateral side	44.1	±	8.0	39.5	±	8.4	0.027
Mid-thigh subcutaneous fat area (cm^2^)								
	Operated side	50.4	±	23.1	61.3	±	31.6	0.126
	Contralateral side	45.7	±	18.6	54.4	±	28.9	0.175
Mid-thigh circumference (cm)								
	Operated side	36.6	±	6.7	39.1	±	5.4	0.132
	Contralateral side	38.6	±	3.3	40.1	±	5.4	0.257
Calf circumference (cm)								
	Operated side	31.5	±	2.0	32.2	±	4.4	0.493
	Contralateral side	31.7	±	2.2	33.0	±	4.6	0.196
Pulmonary function								
	VC (%predicted)	102.2	±	17.4	99.4	±	19.2	0.517
	FEV_1_ (%predicted)	95.1	±	17.3	98.8	±	22.5	0.454
	FEV_1_/FVC	105.9	±	19.5	102.4	±	21.4	0.479
	PEF (%predicted)	89.6	±	16.4	90.6	±	23.8	0.839
Barthel Index (points)		100 [90, 100]			85 [70, 100]			0.002
HADS								
	Anxiety	5	±	3	9	±	5	0.001
	Depression	5	±	4	9	±	5	0.004
JHEQ score (points)								
	Total	26	±	12	20	±	13	0.080
	Pain	10	±	8	9	±	6	0.342
	Function	4	±	4	3	±	4	0.898
	Mental	12	±	5	8	±	6	0.010
JHEQ VAS dissatisfaction scale (mm)		75	±	31	77	±	28	0.760

FEV_1_, forced expiratory volume in 1 s; FVC, forced vital capacity; HADS, hospital anxiety and depression scale; HU, Hounsfield units; JHEQ, Japanese Orthopaedic Association Hip-Disease Evaluation Questionnaire; PEF, peak expiratory flow; VC, vital capacity; VAS, visual analog scale.

**Table A3:** Postoperative physical function and clinical outcomes.

		Overall (n = 254)			Non-Sarcopenia			Sarcopenia			Severe Sarcopenia			*p* Value
					(n = 195; 76.8%)			(n = 32; 12.6%)			(n = 27; 10.6%)			
VAS for pain (mm)														
	Average	8 [3, 15]			7 [2, 14]			11 [7, 18]			8 [2, 13]			0.072
	Maximum	15 [5, 30]			15 [4, 31]			26 [10, 38]			14 [5, 26]			0.090
Hip abductor strength (kgf*m)														
	Operated side	2.5	±	2.6	3.2	±	3.2	1.8	±	1.6 ^a^	1.6	±	0.8 ^a^	0.003
	Contralateral side	3.3	±	2.9	4.3	±	4.1	2.5	±	1.7 ^a^	2.0	±	1.1 ^a^	0.002
Change in hip abductor strength (kgf*m)														
	Operated side	0.0	±	3.5	0.2	±	3.9	−0.7	±	2.3	−0.3	±	1.3	0.450
	Contralateral side	1.0	±	3.5	1.2	±	4.0	0.4	±	1.8	0.2	±	1.2	0.272
Knee extensor strength (kgf*m)														
	Operated side	5.4	±	5.5	6.8	±	6.3	3.4	±	2.1 ^a^	2.6	±	2.2 ^a^	<0.001
	Contralateral side	6.9	±	6.4	9.0	±	7.7	4.8	±	3.6 ^a^	3.7	±	2.7 ^a^	<0.001
Change in knee extensor strength (kgf*m)														
	Operated side	1.3	±	5.3	1.8	±	5.9	0.0	±	2.2	−0.1	±	2.4	0.087
	Contralateral side	2.3	±	6.8	2.8	±	7.7	1.2	±	3.2	0.3	±	2.7	0.138
Handgrip strength (kg)														
	Male	33.2	±	7.9	35.2	±	6.6	25.8	±	11.2 ^a^	22.9	±	2.8 ^a^	<0.001
	Female	21.9	±	5.6	23.6	±	4.9	21.2	±	3.6 ^a^	13.6	±	3.8 ^a,b^	<0.001
Change in handgrip strength (kg)														
	Male	−0.6	±	3.6	−0.5	±	3.2	−0.5	±	7.5	−1.4	±	3.5	0.855
	Female	0.0	±	3.2	−0.1	±	3.4	0.0	±	3.2	0.2	±	1.8	0.938
Usual gait speed (m/s)		0.84	±	0.24	0.92	±	0.25	0.80	±	0.19	0.71	±	0.25 ^a^	<0.001
Change in usual gait speed (m/s)		0.06	±	0.35	0.04	±	0.36	0.11	±	0.36	0.09	±	0.23	0.520
Maximal gait speed (m/s)		1.07	±	0.25	1.17	±	0.29	0.99	±	0.25 ^a^	0.95	±	0.24 ^a^	<0.001
Change in maximal gait speed (m/s)		−0.12	±	0.30	−0.14	±	0.31	−0.09	±	0.29	0.03	±	0.23	0.096
Barthel Index at discharge (points)		100 [95, 100]			100 [95, 100]			100 [95, 100]			95 [83, 100] ^a^			0.022
Change in Barthel Index (points)		0 [−5, 0]			0 [−5, 0]			0 [−5, 0]			0 [−6.3, 0]			0.920
Hospital-acquired disability		72 (28.3)			52 (26.7)			10 (31.2)			10 (37.0)			0.495
Length of hospital stay (day)		17	[15–22]		17	[14–21]		17	[14–24]		21	[17–29] ^a^		0.004

VAS, visual analogue scale. ^a^ Significant difference from the non-sarcopenia group. ^b^ Significant difference from the sarcopenia group.

**Table A4:** Postoperative clinical outcomes.

	Severe Sarcopenia	No Sarcopenia Assessment	*p* Value
	(n = 27)	(n = 52)	
Functional recovery delay on POD7	10 (37.0)	27 (51.9)	0.241
Barthel Index at discharge (points)	95 [85, 100]	80 [75, 100]	0.054
Change in Barthel Index at discharge (points)	0 [−5, 0]	0 [−5, 10]	0.251
Hospital-acquired disability	10 (37.0)	18 (34.6)	1.000
Length of hospital stay (day)	21 [17–29]	21 [16–26]	0.431
Non-home discharge	14 (51.9)	37 (71.2)	0.136

POD, postoperative day.

**Table A5:** Clinical outcomes at 6 months after surgery between two groups.

		Severe Sarcopenia			No Sarcopenia Assessment			*p* Value
		(n = 27)			(n = 52)			
JOA hip score (points)		76	±	17	74	±	17	0.642
JHEQ score (points)								
	Total	50	±	17	51	±	19	0.910
	Pain	23	±	7	23	±	7	0.869
	Function	8	±	7	10	±	7	0.449
	Mental	19	±	7	18	±	8	0.796
JHEQ VAS dissatisfaction scale (mm)		35	±	38	23	±	28	0.232
Adverse events within 180 days after surgery		4 (14.8)			13 (25.0)			0.392

JOA, Japanese Orthopaedic Association; JHEQ, Japanese Orthopaedic Association Hip-Disease Evaluation Questionnaire; VAS, visual analog scale.
